# The Pharmaceutical Pricing and Reimbursement Information (PPRI) network – a decade of exchange of information and policy research?

**DOI:** 10.1186/2052-3211-8-S1-E4

**Published:** 2015-10-05

**Authors:** Sabine Vogler, Nina Zimmermann, Kees De Joncheere

**Affiliations:** 1 WHO Collaborating Centre for Pharmaceutical Pricing and Reimbursement Policies, Health Economics Department, Gesundheit Österreich GmbH (Austrian Public Health Institute), Vienna, 1010, Austria; 2 Essential Medicines and Health Products Department (EMP), World Health Organization (WHO), 1211 Geneva 27, Switzerland

## 

Policy-makers throughout Europe and beyond have been working to develop and implement, and further adjust, the most appropriate mix of policy measures in pharmaceutical pricing and reimbursement to ensure equitable access to medicines despite limited budgets. The experience with policies in other countries is highly valuable information for them.

In order to support policy-makers, the Pharmaceutical Pricing and Reimbursement Information (PPRI) network of competent authorities was established in 2005, with the aim to offer a platform for competent authorities of pharmaceutical pricing and reimbursement to exchange information and data and to establish a sustainable reporting system for country information.

Within the decade of PPRI's existence, more than 60 country reports (information about medicines policies for the out-patient sector, hospital pharma reports, and integrated country profiles about the out-patient and the in-patient sectors) of 28 different countries and more than 60 country posters were produced. Comparative analyses were undertaken, e.g. in the PPRI report [1], the PHIS Hospital Pharma report [2] or in scientific articles [3-5]. A glossary [6] and indicators were developed and are regularly updated. These deliverables have been shared in the open domain (http://whocc.goeg.at/Publications/).

In addition to these reports and tools, the instrument of PPRI network queries has proven its importance. A PPRI network query allows PPRI network members to ask for specific and quick information about a policy and situation in the other countries represented in the PPRI network [7]. In total, 319 PPRI queries have been launched until June 2015.

The PPRI network is predominantly Europe-based. It has been growing over the years and currently comprises competent authorities for pharmaceutical pricing and reimbursement from 45 countries (thereof all 28 European Union Member States, another twelve European countries, and five non-European countries) as well as European and international institutions (European Commission, OECD, WHO, World Bank). Face-to-face meetings of the PPRI network are organised twice a year. The PPRI network is characterized by trust and mutual respect.

The last decade has brought both new challenges (e.g. financial constraints due to the crisis, new premium-priced medicines) and opportunities (e.g. new policy tools such as managed-entry agreements, patent expiries of biotechnological medicines) for policy-makers. Participation in the PPRI network has supported them to deal with these developments and discuss possible solutions and collaborative approaches. In the light of on-going challenges, the PPRI network is well placed and prepared to continue playing its role as discussion and information exchange platform for the years to come.

## References

[B1] Vogler S Habl C Leopold C Rosian-Schikuta I de Joncheere K Lyager Thomsen T PPRI Report 2008 Vienna: Pharmaceutical Pricing and Reimbursement Information http://whocc.goeg.at/Literaturliste/Dokumente/BooksReports/PPRI_Report_final.pdf. Accessed 15 July 2015

[B2] Vogler S Habl C Leopold C Mazag J Morak S Zimmermann N PHIS Hospital Pharma Report 2010 Vienna: Pharmaceutical Health Information System (PHIS) http://whocc.goeg.at/Literaturliste/Dokumente/BooksReports/PHIS_Hospital%20Pharma_Report.pdf. Accessed 15 July 2015

[B3] Vogler S Espin J Habl C Pharmaceutical Pricing and Reimbursement Information (PPRI) – New PPRI analysis including Spain Pharmaceuticals, Policy and Law 2009 11 3 213 234

[B4] Vogler S Habl C Bogut M Voncina L Comparing pharmaceutical pricing and reimbursement policies in Croatia to the European Union Member States Croatian Medical Journal 2011 52 2 183 197 21495202 10.3325/cmj.2011.52.183 PMC3081217

[B5] Vogler S The impact of pharmaceutical pricing and reimbursement policies on generics uptake: implementation of policy options on generics in 29 European countries—an overview Generics and Biosimilars Initiative Journal 2012 1 2 93 100

[B6] WHO Collaborating Centre for Pharmaceutical Pricing and Reimbursement Policies Glossary of pharmaceutical terms. Update 2013 2013 Vienna http://whocc.goeg.at/Literaturliste/Dokumente/MethodologyTemplate/PHIS%20Glossary_Update2013_final_gesamt.pdf

[B7] Vogler S Leopold C Zimmermann N Habl C de Joncheere K The Pharmaceutical Pricing and Reimbursement Information (PPRI) initiative–experiences from engaging with pharmaceutical policy makers Health Policy and Technology 2014 3 2 139 148

